# Reduction of Antibiotic Prescription in Complicated Appendicitis through Behavioral Change Measures

**DOI:** 10.1055/s-0044-1801350

**Published:** 2025-01-08

**Authors:** Alberto A. Artiles Garcia, Humberto Guanche Garcell, Miguel J. Pinto Echevarría, Carlos A. Sanchez Portela, Carlos M. Sanchez Rivas, Marlon Arias Medina, Niuvis Seoane Perez, Osiris I. Escobar More, Tania M. Fernandez Hernandez

**Affiliations:** 1 Department of Surgical, The Cuban Hospital, Dukhan, Doha, Qatar; 2 Department of Infection Control, The Cuban Hospital, Dukhan, Doha, Qatar; 3 Depártment of Pharmacy, The Cuban Hospital, Dukhan, Doha, Qatar; 4 Depártment of Medical Administration, The Cuban Hospital, Dukhan, Doha, Qatar

**Keywords:** appendicitis, complicated, antibiotics, intervention, cost, behavioral change

## Abstract

**Background** Variability in the prescription of antibiotics constitutes an area for improvement related to patient safety issues, including the risk of infection and health care efficiency based on evidence.

**Objectives** The study aims to evaluate the effect of an intervention to reduce the duration of antibiotic treatment in complicated appendicitis.

**Methods** A quality improvement program was implemented in the surgical department of The Cuban Hospital (Doha, Qatar). During a 3-month baseline period, data about antibiotic duration, consumption (daily defined doses), and cost (in Qatary Riyals) were identified, as well as during two plan-do-study-act (PDSA) intervention periods. Interventions include action focused on staff education, monitoring antibiotic use, feedback, and reminders during discharge planning.

**Results** At baseline, 13 patients with complicated appendicitis were documented, while there were 41 and 15 patients during PDSA cycles 1 and 2, respectively. A 29.5% reduction in days of antibiotic treatment was observed during the PDSA cycle 2 in comparison with the baseline. Accordingly, a reduction of 38.7% in the consumption of antibiotics and a reduction of 24.6% in cost were observed, with no adverse outcomes for patients during the 30-day follow-up period.

**Conclusion** The intervention resulted in an improvement in antibiotic use with satisfactory patient outcomes and an additional effect on the efficiency of health care and the prevention of microbial resistance and other adverse effects.

## Introduction

Variability in health care practices is frequently observed and is sometimes associated with failures in compliance with evidence-based recommendations. When this is linked to unnecessary or excessive antibiotics use for the management of selected clinical conditions, it has additional implications that include adverse effects (e.g., *Clostridium difficile* infections), the development of antimicrobial resistance, and increased cost among others. 1 2

In complicated appendicitis, antibiotics are required after the surgical procedure, and various studies described proper outcomes with short duration of antibiotics treatment in complicated appendicitis. 3 4 The recent guidelines of the World Society of Emergency Surgery recommend limiting antibiotics use to 3 to 5 days if adequate source control is achieved. 5

During 2022, we conducted a quality improvement (QI) intervention to improve antibiotic prophylaxis in various surgical procedures and observed a significant variability of antibiotic treatment in complicated appendicitis. 6 Patients with an intraoperative diagnosis of gangrenous, perforated, or appendicular mass received, in addition to the surgical prophylaxis, up to 11 days of antibiotics mainly cefuroxime plus metronidazole as per hospital policy.

Accordingly, from the previous findings, we decide to develop a QI intervention to reduce the duration of the antibiotic course in complicated appendicitis by 25% from baseline by December 2023.

## Materials and Methods

A QI program was implemented in the surgical department of The Cuban Hospital, a community hospital member of Hamad Medical Corporation (Doha, Qatar). The project was approved by the Quality and Patient Safety Department and recorded in Life QI ( https:/lifeqisystem.com ).

The QI project team includes members of the surgical team, pharmacy, and infection control department. The Corporate Policy CL 7197 on antimicrobial prescribing recommends the use of cefuroxime plus metronidazole for surgical prophylaxis.

Complicated appendicitis includes the appendicular mass, gangrenous, and perforated according to surgeon's assessment during the surgical procedures (most performed through laparoscopic methods). Baseline data collection was conducted over a 3-month period including days on antibiotics (during admission plus after discharge), antibiotic consumption using Defined Daily Dose (DDD)/Anatomical Therapeutic Chemical (ATC) system (ATC/DDD), and antibiotic cost (as per hospital prices in Qatari Riyals [QR]). No action for improvement was implemented during baseline including staff feedback.

### Intervention

The baseline period includes 13 patients with complicated appendectomies during January to March 2023. After the baseline data collection from the patients' electronic medical records, the surgical team and key leaders were informed about the summary of baseline data to support further actions focused on staff education, monitoring antimicrobial use, and feedback to staff.

Two plan-do-study-act (PDSA) cycles were conducted ( Table 1 ). The first PDSA cycle (April–August 2023, 41 patients) includes a dedicated session review with the surgical team of the current recommendation for antibiotic prophylaxis and treatment of complicated appendicitis. The monitoring of antibiotic prescription duration was performed weekly and the staff was informed through a progress report distributed by email, and open analysis during morning meetings was done. During the second PDSA cycle (September–December 2023, 15 patients), additional data analysis of patients with prolonged antibiotic use was performed including its rationality based on evidence. The close monitoring of antibiotic duration and timely feedback to staff were implemented including the analysis during the discharge planning. The discharge planning is performed usually the day before discharge and includes communication with the patient, the review of the postdischarge management, and patient and family educational needs.

**Table 1:** Description of PDSA cycles

	PDSA cycle 1	PDSA cycle 2
Time	April–August 2023	September–December 2023
Plan	1. To review and evaluate with the surgical team, the current recommendations of antibiotic prophylaxis based on international guidelines and corporate policies 2. To decide on the appropriate prescription according to intraoperative findings 3. To evaluate the required duration (maximum 5–7 d) during the morning team meeting and at discharge	1. To conduct a detailed analysis of prolonged antibiotic prescriptions done patient by patient based on the supporting evidence 2. To review the antibiotic prescriptions by a clinical pharmacist
Do	1. Review the appropriateness of prescription during morning team meetings and at discharge 2. Weekly monitoring of the duration of antibiotic prescription 3. Weekly report to staff and analysis	1. Daily review of antibiotic prescription at discharge by the surgical team 2. Weely monitoring maintained
Study		*Measure 1* . The mean duration of antibiotic prescription. Formula: sum of prescribed days on antibiotics (during admission plus after discharge) divided by the number of patients with complicated appendicitis *Measure 2* . Antibiotic consumption. Formula: Sum of daily defined doses of antibiotic prescribed by 100 procedures *Measure 3* . Antibiotic cost. Formula: Sum of cost of antibiotic prescribed by 100 procedures Data recorded in an Excel sheet	
Act	- Reduction in duration of antibiotic prescription - Staff involvement	- Improved prescription practices - Reduction of antibiotic consumption and related cost - Further analysis to identify action for sustainability is required

Abbreviation: PDSA, plan-do-study-act.

The Revised Standards for Quality Improvement Reporting Excellence (version 2.0) were followed in this QI project.

Analysis of data was performed using descriptive statistics. The antimicrobial consumption and cost were presented as DDD and QR per 100 patients, respectively.

## Results

At baseline, 13 patients were documented with complicated appendicitis (13/70 appendectomies, 18.6%), while 41 (41/175 appendectomies, 23.4%) and 15 patients (15/108 appendectomies, 13.9%) during PDSA cycles 1 and 2, respectively. Appendicular mass was reported in 36 patients, gangrenous appendicitis in 30 patients, and perforated appendicitis in 3 patients.

**Fig. 1:**
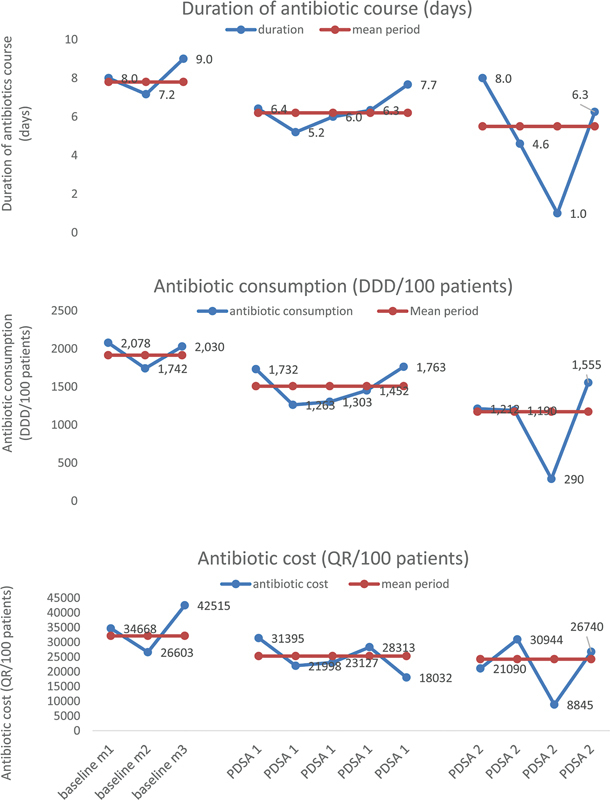
Duration of antibiotic course, antibiotic consumption, and cost in complicated appendicitis (days) during baseline and intervention period. DDD, Daily Defined Dose; QR, Qatari riyals.

The mean duration of antibiotic treatment was 7.8 days at baseline, and 6.2 and 5.5 days during PDSA cycles 1 and 2, with a 29.5% reduction during the intervention, achieving the QI project goal. The mean antimicrobial consumption was 1,915 DDD/100 patients at baseline and 1,508 and 1,173 DDD/100 patients during PDSA cycles 1 and 2 with a 38.7% reduction. The antimicrobial cost was 32,153 QR/100 patients at baseline, and 25,285 and 24,249 QR/100 patients during PDSA cycles 1 and 2 with a 24.6% reduction ( Fig. 1 ).

**Fig. 2:**
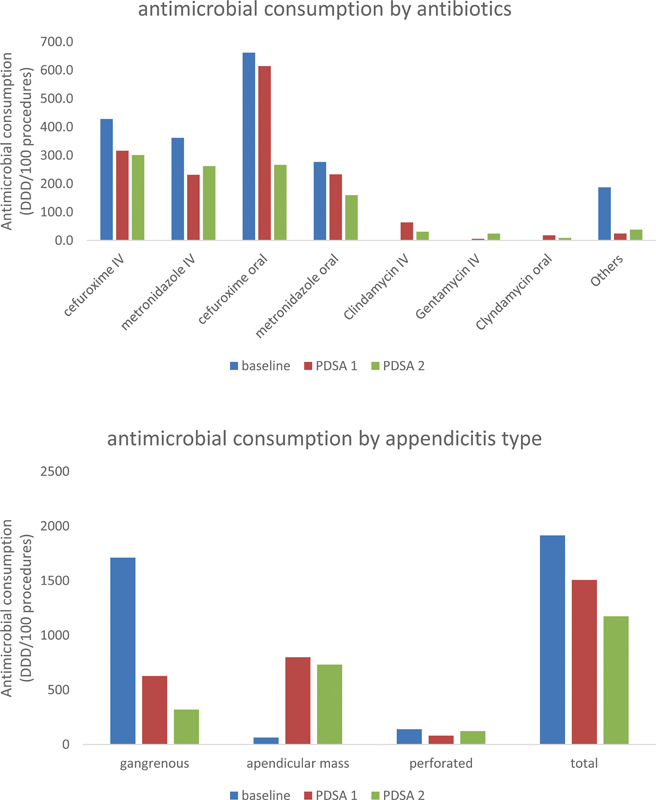
Antimicrobial consumption by antibiotic type and appendicitis type according to periods (DDD/100 procedures). DDD, Daily Defined Dose; IV, intravenous; PDSA, plan-do-study-act.

The reduction of duration (23%) and antimicrobial consumption (40.4%) was mainly in patients with gangrenous or perforated appendicitis, while the cost reduction was higher in appendicular mass (30.1%) ( Fig. 2 ). The combination of cefuroxime–metronidazole was more than 90% of antibiotic consumption during baseline and PDSA cycle 1, and in PDSA cycle 2, this was reduced to 84% due to its limited availability and the use of alternative prophylaxis (gentamycin plus clindamycin). Therefore, the combination of cefuroxime–metronidazole contributes with 93, 87, and 85% of the cost during the baseline and PDSA cycles 1 and 2, respectively ( Fig. 2 ). The mean duration of inpatient treatment was 2.8 days and after discharge was 4.3 days by switching from intravenous to oral route.

All patients were followed up 30 days after the procedure date and no complication was detected including surgical site infections.

## Discussion

The study has shown the reduction of antibiotic treatment after complicated appendicitis by an intervention focused on increasing the staff's awareness about the need for the rational use of antibiotics based on the best evidence available. 5 7

Regardless of the observed reduction in antibiotic use, we consider potential additional reduction could be achieved, in gangrenous or perforated appendicitis, mainly in patients with limited evidence of disseminated or severe abdominal infection observed during the surgical procedure.

Laverde et al, in a cohort study, reported that an extended duration of antibiotic treatment was not associated with a reduced risk of developing surgical infection after appendectomy. 8 Other studies report similar findings in adults and pediatric patients. 9 10

While the ATC/DDD system is valuable for monitoring antibiotic consumption and its impact on health care efficiency, the guidelines recommend the maximum duration of treatment in complicated appendicitis. 5 11 The Access, Watch, Reserve (AWaRe) guideline indicates that in complicated appendicitis, antibiotic therapy can be continued for 5 days if the symptoms have resolved and the source of the infection has been eliminated with surgery. 11

Actions to promote behavioral change in prescription practices have demonstrated the improvement in antibiotic practices in appendicitis and other clinical conditions. 12 13 In addition to the education, monitoring, and feedback to staff, reminders during the discharge planning could be important. The discharge planning is a key moment to review the prescription practices and identify the required postdischarge antibiotics.

The most significant challenge is the sustainability of the practices in health care settings with frequent staff renewal. Then, long-term sustainability requires continuous staff education, up-to-date antimicrobial policy based on the best evidence about the duration of antibiotic treatment in complicated appendicitis, and monitoring and feedback to staff.

*Study limitation* : In a single-center experience with a low volume of surgical procedures, implementing QI initiatives could be easier, and staff communication could be more expedited.

## Conclusion

The QI intervention resulted in a reduction in the duration of antibiotic treatment in patients with complicated appendicitis, with satisfactory patient outcomes. Additionally, the reduction in consumption and costs of antimicrobial use impacted the efficiency of health care and the prevention of microbial resistance and other adverse effects.
